# Under the Influence: Alcohol, Substance Use and Treatment Adherence in Youth Living with HIV in the Global Adolescent and Young Adult Network of IeDEA (AYANI) Cohort

**DOI:** 10.1007/s10461-026-05127-0

**Published:** 2026-04-21

**Authors:** Nicola van Dongen, Karl-Günter Technau, Florence Jaguga, Gad Murenzi, Daisy M. Machado, Madeleine Amorissani-Folquet, Rossana Ditango, Fiona Mureithi, Brittany McCoy, Ellen Brazier, Marco Tulio Luque, Julie Jesson, Thanyawee Phuthanakit, Nicola Maxwell, Rachel Vreeman, Batya Elul, Jorge A. Pinto, Gladis Oka, Tavitiya Sudjaritruk, Tukiya Kanguya, Leslie Enane, Diane Ryumugabe, Genevieve Hilaire, Valériane Leroy, Annette H. Sohn, Aaron Brehm, Kim Anderson

**Affiliations:** 1 Empilweni Services and Research Unit, Department of Paediatrics and Child Health, Faculty of Health Sciences, Rahima Moosa Mother and Child Hospital, University of the Witwatersrand, Johannesburg, South Africa; 2 Alcohol & Drug Abuse Rehabilitation Services, Moi Teaching & Referral Hospital, Eldoret, Kenya; 3 Academic Model Providing Access to Healthcare (AMPATH) Kenya, Eldoret, Kenya; 4 Research for Development and Rwanda Military Referral and Teaching Hospital, Kigali, Rwanda; 5 Department of Pediatrics, Pediatric Infectious Diseases Division, Escola Paulista de Medicina-Universidade Federal de São Paulo (UNIFESP), São Paulo, Brazil; 6 University hospital Cocody, Abidjan, Côte d’Ivoire; 7 Research Institute for Tropical Medicine, Manila, Philippines; 8 Centre for Infectious Disease Research in Zambia, Lusaka, Zambia; 9 Department of Psychiatry, Department of Global Health and Health System Design, Icahn School of Medicine at Mount Sinai, New York, NY USA; 10 Institute for Implementation Science in Population Health, City University of New York, New York, NY USA; 11 Servicio de Infectología, Departamento de Pediatría, Servicio de Infectología, Hospital Escuela, Instituto Hondureño de Seguridad Social, Tegucigalpa, Honduras; 12 CERPOP, Inserm, Toulouse University, Toulouse, France; 13 Department of Pediatrics, Faculty of Medicine, Chulalongkorn University, Bangkok, Thailand; 14 Centre for Integrated Data and Epidemiological Research, School of Public Health, University of Cape Town, Cape Town, South Africa; 15 Department of Global Health and Health Systems Design, Icahn School of Medicine at Mount Sinai, Arnhold Institute for Global Health, New York, NY USA; 16 Department of Epidemiology, Mailman School of Public Health, Columbia University, New York, NY USA; 17 Department of Pediatrics, School of Medicine, Federal University of Minas Gerais, Belo Horizonte, Brazil; 18 Department of Pediatrics, and Clinical and Molecular Epidemiology of Emerging and Re-emerging Infectious Diseases Research Cluster, Faculty of Medicine, Chiang Mai University, Chiang Mai, Thailand; 19 The Ryan White Center for Pediatric Infectious Disease and Global Health, Department of Pediatrics, Indiana University School of Medicine, Indianapolis, USA; 20 Les Centres GHESKIO, Port-au-Prince, Haïti; 21 TREAT Asia/amfAR, The Foundation for AIDS Research, Bangkok, Thailand; 22 Department of Biostatistics and Health Data Science, Indiana University School of Medicine, Indianapolis, IN USA

**Keywords:** HIV, Adolescents, Substance use, Adherence, Mental health

## Abstract

Alcohol and substance use in youth living with HIV (YLHIV) can influence health and treatment outcomes. We assessed substance use and associations with antiretroviral therapy (ART) adherence among YLHIV. The Adolescent and Young Adult Network of IeDEA enrolled YLHIV (15–24 years old) receiving ART (2021–2024) from 6 global regions. We assessed biopsychosocial experiences with validated tools; adherence with self-reported missed medication doses and unsuppressed viral load (VL > 1,000 copies/ml); and substance use by point-of-care urine tests and Alcohol, Smoking and Substance Involvement Screening Test (ASSIST) tool. Among 694 YLHIV, 53% female, median enrolment 20 years old (IQR 18–22), 83% had perinatally-acquired HIV, and 71% received Integrase Inhibitor-based ART. Half (49%) reported missing medication doses and 14% had unsuppressed VL. Any prior alcohol use (47%) was more common than other substances, where cannabis (12%) and sedatives/benzodiazepines (3.9%) contributed. Recent moderate/high-risk alcohol and other substance use were reported by 9.1% of YLHIV. YLHIV with positive urine tests had not reported recent use of alcohol (*n* = 17/46; 37%), cannabis (*n* = 12/32; 38%) and benzodiazepines (*n* = 13/13, 100%). Moderate/high-risk alcohol and other substance use were associated with self-reported adherence difficulties (adjusted odds ratio(aOR) 2.37, 95% CI 1.28–4.38; and aOR 2.13, 95% CI 1.18–3.85) but not unsuppressed VL (aOR 0.80, 95% CI 0.34–1.88; and aOR 1.61, 95% CI 0.79–3.29). Moderate/high-risk alcohol and/or substance use was reported by 15% of YLHIV and was associated with self-reported adherence difficulties. Discrepancies between point-of-care tests and self-reported use suggest alcohol/substance use is underestimated. Enhanced screening and interventions targeting substance use are recommended.

## Background

In 2023, there were 3.2 million youth living with HIV (YLHIV, age 15–24 years), representing 8% of all people living with HIV [1]. Adolescents often have worse treatment outcomes compared to adults, including poorer retention in care, adherence and viral suppression [2–4]. Furthermore, youth with perinatally-acquired HIV may have poorer outcomes than those with non-perinatally-acquired HIV [2, 5, 6]. The development of well-tolerated regimens has reduced morbidity and mortality and improved quality of life. However, ART adherence among YLHIV may be affected by a myriad of factors as they transition into adulthood [7]. HIV-related factors such as disclosure status, duration of treatment, treatment fatigue, and treatment regimen may influence adherence. Biopsychosocial factors may also play a role, such as gender, poverty, incarceration, stigma, fear of rejection, exposure to trauma, substance use, mental health disorders, reduced parental support and adverse life events [2, 6, 8–16]. Addressing biopsychosocial factors is important for optimising care and outcomes for YLHIV.

Substance use disorders often begin in adolescence and constitute a leading health risk for adolescents and young adults globally [7, 17, 18]. Most adolescents’ experience substance use debut between 13 and 15 years old and maximum experimentation between 18 and 25 years, often because of curiosity, peer pressure or poor self-esteem [19, 20]. For about 60–80% of all adolescents who use substances, use is limited to the adolescent period [19, 20]. Studies have found that substance use among YLHIV may be high [2, 4]. Most studies on substance use in YLHIV are from high-income countries, studies around substance use among YLHIV from resource-limited settings are scarce and show varying results. A US study showed alcohol use in 21% and cannabis use in 28% of YLHIV [13]. In a study from Mexico, around 20% of adolescents living with HIV had used any substance, whereas almost all had tried alcohol at least once [9]. In contrast, a study of YLHIV from Tanzania found relatively low prevalence of substance use (7%) [21]. 

The same stressors that can influence adherence to ART, linkage to care, and general health outcomes may also influence experimentation with substances, though the mechanisms of interaction are not clearly defined [7, 13]. Living in chaotic situations with little support and high exposure to trauma may make it difficult to take ART and may make substance use more attractive [6]. Alternatively, using substances might impact executive functioning or make one less likely to remember medication [22–24]. 

Substance use has been associated with increased high-risk sexual activity, poor ART adherence, lack of viral suppression, drug resistance and poorer clinical outcomes than among those who do not use substances [6, 13, 20, 25–27]. YLHIV with problematic substance use are three times more likely to report poor ART adherence than those without substance use [25]. Additionally, anxiety and depression are common in adolescents living with HIV [2, 18], with YLHIV having an increased burden of mental health difficulties, particularly among those with perinatally-acquired HIV [15]. In turn, use of substances, especially tobacco and cannabis, may increase when depression or anxiety is present, and poor mental health may increase the risk of non-adherence to treatment [9, 15, 28]. 

Given the limited data on YLHIV in resource-limited settings, this study primarily aimed to assess substance use and its associations with adherence through adjusted analyses, in a diverse, predominantly resource-limited cohort. As a secondary aim, associations between substance use, adherence, and other biopsychosocial factors were explored using unadjusted analyses.

## Methods

The Adolescent and Young Adult Network of IeDEA (AYANI) is a global cohort study examining clinical, psychological and social factors that impact HIV care outcomes among YLHIV. AYANI covers 16 sites across six IeDEA regions: East Africa (4 sites in Kenya), Central Africa (2 sites in Rwanda), West Africa (1 site in Cote d’Ivoire), Southern Africa (1 site in South Africa, 1 in Zambia), Asia-Pacific (2 sites in Thailand, 1 in the Philippines) and Caribbean, Central and South America (CCASAnet) (1 site in Honduras, 1 in Haiti, 2 in Brazil) [29]. Sites were selected to meet the requirements of target enrolment numbers and distribution of HIV exposure category (minimum 60% perinatally-acquired HIV) while accounting for feasibility issues (such as existing research infrastructure available at the above IeDEA sites). Sites were in urban (*n* = 14) or peri-urban (*n* = 2) locations [30]. 

YLHIV were enrolled from 2021 to 2024 to provide in-depth data on biological, psychological and social factors experienced. Inclusion criteria for YLHIV included: age 15–24 years; having full knowledge of their HIV diagnosis; enrolled in care at one of the study sites and having had at least one clinic visit in the six months prior to study enrolment and receipt of ART for a minimum of three months prior to enrolment, as per routine clinic records, without further specification regarding treatment interruptions. Informed consent, or a combination of assent and parental/guardian consent for adolescent minors, was obtained as required by participating site institutional review boards or ethics committees. The initial/enrolment study visit included a comprehensive, structured assessment that encompassed several standardised data collection tools. In this study, we conducted a cross-sectional analysis of participant enrolment data.

### Adherence

Adherence was assessed using two separate measures: (a) self-reported adherence using the IeDEA Comprehensive Adherence Measure for Paediatrics (ICAMP) questionnaire [31, 32], and (b) viral load (VL) results collected from routine clinical data. Two ICAMP items were used to capture recent adherence behaviour: one assessing the number of days with at least one missed dose in the preceding 7 days, and a separate item assessing whether any medication doses were missed in the preceding month. Participants were classified as having self-reported adherence difficulties if they reported any missed doses on either ICAMP item. Unsuppressed VL as was defined as > 1,000 copies/ml.

### Alcohol, Tobacco and Substance Use

Participants completed the Alcohol, Smoking and Substance Involvement Screening Test (ASSIST) and the Alcohol Use Disorders Identification Test-10 (AUDIT-10a) [32], validated tools for self-reported use of alcohol, tobacco and other substances (e.g. sedatives, cocaine, amphetamines, hallucinogens) [33, 34]. 

The ASSIST tool captures (a) lifetime substance use (‘ever used’ a substance), (b) frequency of recent use (defined as in the last three months), and (c) level of substance-related risk. ASSIST scores > 10 for alcohol indicate recent moderate/high risk use (requiring intervention), whereas for other substances, ASSIST scores > 3 indicate recent moderate/high risk. Those with moderate risk scores are at risk of current and future health and social problems related to substance use, including the possibility of dependence, and suggests that, at a minimum, a brief intervention (discussion) is warranted. Those with high-risk scores are at high risk of dependence or are substance-dependent and may be likely to experience health, social, financial, legal and relationship problems because of their substance use. Referral to specialist assessment and treatment is warranted [34]. 

AUDIT scores ≥ 8 indicate a strong likelihood of hazardous or harmful alcohol consumption in the last year [33]. Urine point-of-care tests for alcohol, cannabis, opioids, benzodiazepines and amphetamines were performed, providing a positive or negative result. Urine point-of-care tests used were determined by the study site based on the available test kits in the region. Typically, urine tests will be positive if substances were used within 72 h prior to the test. Cannabis metabolites may be detected in urine for 2 weeks or longer, depending on the sensitivity of the test and the patient’s pattern of use [35]. 

### Mental Health Domains

Aspects of mental health were assessed using standardised tests: Patient Health Questionnaire (PHQ-9) for depression; Generalized Anxiety Disorder 7-item (GAD-7) scale for anxiety; Stigma Assessment for Families Inventory (SAFI) tool for HIV stigma; Life Event Checklist (LEC-5) for potentially traumatic events experienced or witnessed; and Multidimensional Scale of Perceived Social Support (MSPSS) for perceived social support [36–43]. PHQ score ≥ 10 indicates moderate/severe depression; GAD- 7 scores ≥ 10 indicates moderate/severe anxiety; answering positively to any SAFI questions indicates experiencing stigma or discrimination due to HIV; a non-zero LEC-5 score indicates experiencing/witnessing a potentially traumatic event; and MSPSS score < 3 indicates social support perceived as low.

### Statistical Analyses

**Fig. 1:**
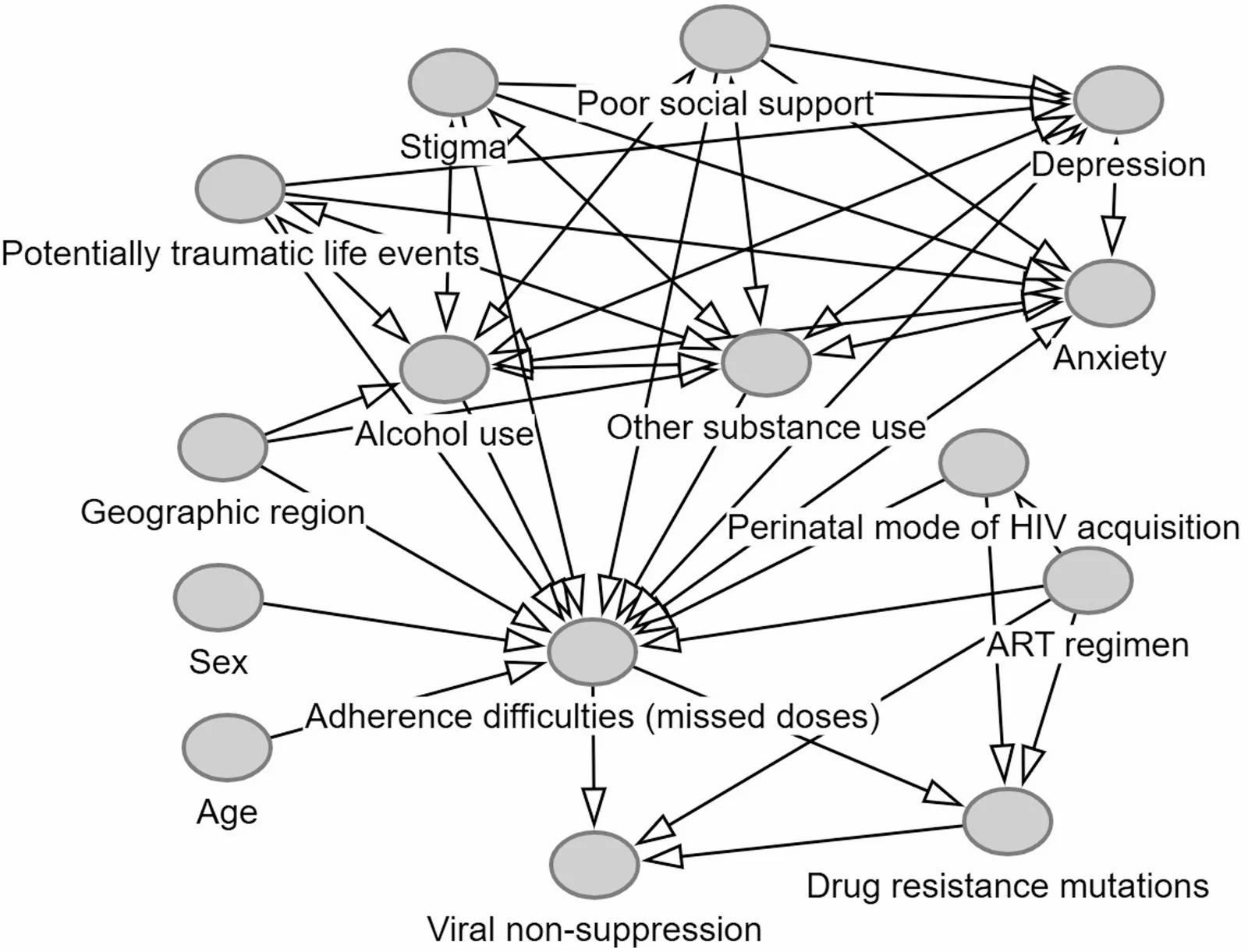
Conceptual Framework

Study data were captured into REDCap electronic data tools hosted at Indiana University, and the data were exported for analysis in Stata 17 (StataCorp. 2021) [44, 45]. We described characteristics of YLHIV using counts and proportions, or medians with interquartile ranges (IQR), as appropriate. We used univariable logistic regression to explore associations between several factors and (a) self-reported adherence difficulties (missed doses) and (b) unsuppressed VL (> 1,000 copies/ml). We also used univariable logistic regression to explore factors associated with moderate/high risk alcohol use and other substance use (separately). We used multivariable logistic regression to assess associations between moderate/high risk alcohol use (ASSIST tool) and other substance use (separately) with (a) self-reported adherence difficulties and (b) unsuppressed VL. We adjusted for sex, age, mode of HIV acquisition, type of ART regimen (defined by anchor drug class) and geographical region. We conducted a sensitivity analysis using a lower VL threshold (> 200 copies/ml) to assess whether associations between substance use and elevated VL differed when using a more sensitive definition of elevated VL. In additional sensitivity analyses, alternative definitions of self-reported adherence difficulties were explored using the ICAMP 7-day item, categorising participants as having missed 0–1 versus ≥ 2 days with missed doses, and 0–3 versus ≥ 4 days with missed doses, to assess whether associations differed with increasing frequency of missed doses. Models were not adjusted for factors that were considered potentially closely correlated with alcohol/substance use (see Fig. 1: conceptual framework to inform analysis, rather than to establish causal relationships). In regression analyses, we excluded tobacco and hookah use from ‘other substance use’ as tobacco is usually considered separately and hookah contents were not specified.

## Results

We included enrolment data for 694 YLHIV: median age at enrolment was 20 years (IQR 18–22); 53% were female; and 83% had perinatally-acquired HIV. Characteristics of enrolled YLHIV are shown in Table 1, stratified by region of enrolment. Overall, 49% reported missing any medication doses in the previous month and 14% had unsuppressed VL. Among participants with available ICAMP data, most adherence lapses were infrequent: 61% reported no missed doses in the previous 7 days, 19% missed doses on one day, 16% on two or three days, and 4% reported missing doses on four or more days. Among participants with available VL data, 83% of measurements were obtained on the day of study enrolment and 98% within three months of enrolment. Most participants (71%) were prescribed integrase strand transfer inhibitor (INSTI)-based regimens (dolutegravir in 99% of these). Moderate/severe anxiety and depressive symptoms were each reported by 10% of participants, with 15% reporting either condition.

**Table 1:** Characteristics of 694 youth living with HIV

		Total	Region					
			EA	SA	CA	WA	CN	AP
Number of participants		694 (100%)	197 (28.4%)	100 (14.4%)	128 (18.4%)	83 (12.0%)	111 (16.0%)	75 (10.8%)
Sex^a^ (*n* = 694)	Male	327 (47.1%)	85 (43.1%)	48 (48.0%)	51 (39.8%)	40 (48.2%)	53 (47.7%)	52 (69.3%)
	Female	365 (52.6%)	112 (56.9%)	52 (52.0%)	77 (60.2%)	43 (51.8%)	58 (52.3%)	23 (30.7%)
Age at enrollment, median IQR (years) (*n* = 694)		20.0 (18.0-22.2)	19.7 (17.0-22.1)	18.9 (17.3–20.8)	20.8 (18.7–23.2)	18.7 (17.3–20.3)	21.7 (19.5–23.3)	21.1 (19.1–23.7)
Age category at enrollment (years) (*n* = 694)	15–17	172 (24.8%)	66 (33.5%)	33 (33.0%)	26 (20.3%	28 (33.7%)	8 (7.2%)	11 (14.7%)
	18–19	177 (25.5%)	42 (21.3%)	34 (34.0%)	27 (21.1%)	34 (41.0%)	25 (22.5%)	15 (20.0%)
	20–21	154 (22.2%)	39 (19.8%)	26 (26.0%)	25 (19.5%)	14 (16.9%)	28 (25.2%)	22 (29.3%)
	22–25	191 (27.5%)	50 (25.4%)	7 (7.0%)	50 (39.1%)	7 (8.4%)	50 (45.0%)	27 (36.0%)
Perinatally acquired HIV (vs. non-perinatally acquired) (*n* = 693)		575 (83.0%)	163 (82.7%)	98 (98.0%)	100 (78.1%)	80 (97.6%)	95 (85.6%)	39 (52.0%)
Currently schooling (*n* = 690)	Yes (vs. no)	408 (59.1%)	114 (58.5%)	75 (75.0%)	69 (53.9%)	61 (73.5%)	45 (40.5%)	44 (60.3%)
Highest schooling level to date (*n* = 694)	None	11 (1.6%)	1 (0.5%)	0	8 (6.3%)	0	1 (0.9%)	1 (1.3%)
	Primary school	125 (18.0%)	39 (19.8%)	7 (7.0%)	60 (46.9%)	8 (9.6%)	9 (8.1%)	2 (2.7%)
	Secondary school	425 (61.2%)	123 (62.4%)	77 (77.0%)	45 (35.2%)	64 (77.1%)	69 (62.2%)	47 (62.7%)
	Technical/vocational training institution/College/Pre University	73 (10.5%)	22 (11.2%)	13 (13.0%)	11 (8.6%)	1 (1.2%)	10 (9.0%)	16 (21.3%)
	University	51 (7.4%)	12 (6.1%)	3 (3.0%)	0	10 (12.1%)	20 (18.0%)	6 (8.0%)
	Other	9 (1.3%)	0	0	4 (3.1%)	0	2 (1.8%)	3 (4.0%)
Source of income/support (*n* = 686)	Dependent on someone else (vs. self-supported)	527 (76.8%)	157 (80.1%)	87 (89.7%)	97 (75.8%)	75 (92.6%)	67 (60.9%)	44 (59.5%)
Currently in a relationship (*n* = 688)	Yes (vs. no)	251 (36.5%)	67 (34.4%)	35 (35.4%)	58 (45.3%)	27 (32.5%)	42 (38.2%)	22 (30.1%)
Primary caregiver (*n* = 694)	Self	180 (25.9%)	30 (15.2%)	30 (30.0%)	33 (25.8%)	15 (18.1%)	55 (49.6%)	17 (22.7%)
	Mother	269 (38.8%)	75 (38.1%)	43 (43.0%)	66 (51.6%)	31 (37.4%)	31 (27.9%)	23 (30.7%)
	Father	70 (10.1%)	25 (12.7%)	6 (6.0%)	8 (6.3%)	19 (22.9%)	3 (2.7%)	9 (12.0%)
	Aunt/uncle/grandparent	103 (14.8%)	38 (19.3%)	16 (16.0%)	8 (6.3%)	15 (18.1%)	10( 9.0%)	16 (21.3%)
	Sibling	25 (3.6%)	8 (4.1%)	2 (2.0%)	6 (4.7%)	3 (3.6%)	3 (2.7%)	3 (4.0%)
	Spouse/partner	16 (2.3%)	6 (3.1%)	0	5 (3.9%)	0	1 (0.9%)	4 (5.3%)
	Other	31 (4.5%)	15 (7.6%)	3 (3.0%)	2 (1.6%)	0	8 (7.2%)	3 (4.0%)
Ever been homeless (*n* = 692)		65 (9.4%)	15 (7.6%)	4 (4.0%)	27 (21.1%)	3 (3.6%)	11 (9.9%)	5 (6.7%)
Ever been imprisoned (*n* = 693)		32 (4.6%)	18 (9.1%)	2 (2.0%)	9 (7.0%)	0	3 (2.7%)	0
Any self-reported general difficulties with medication adherence^b^ (*n* = 694)		539 (77.7%)	114 (57.9%)	81 (81.0%)	120 (93.8%)	76 (91.6%)	92 (82.9%)	56 (74.7%)
Self-reported difficulties with medication adherence, assessed by any missed medication doses in the previous month^c^ (*n* = 694)		342 (49.3%)	68 (34.5%)	54 (54.0%)	92 (71.9%)	46 (55.4%)	62 (55.9%)	20 (26.7%)
Perceived social support^d^ (*n* = 694)	Low	54 (7.8%)	15 (7.6%)	5 (5.0%)	20 (15.6%)	3 (3.6%)	8 (7.2%)	3 (4.0%)
	Moderate	303 (43.7%)	80 (40.6%)	31 (31.0%)	81 (63.3%)	31 (37.4%)	45 (40.5%)	35 (46.7%)
	High	337 (48.6%)	102 (51.8%)	64 (64.0%)	27 (21.1%)	49 (59.0%)	58 (52.3%)	37 (49.3%)
Experienced stigma or discrimination due to HIV^e^ (*n* = 691)	Yes (vs. no)	548 (79.3%)	132 (67.7%)	88 (88.9%)	114 (89.1%)	51 (61.5%)	101 (91.0%)	62 (82.7%)
Strong likelihood of hazardous/harmful alcohol consumption^f^ (*n* = 689)	Yes (vs. no)	75 (10.9%)	8 (4.1%)	8 (8.0%)	24 (18.8%)	8 (10.3%)	15 (13.5%)	12 (16.0%)
Moderate/high risk for alcohol use^g^ (*n* = 689)		63 (9.1%)	4 (2.0%)	11 (11.0%)	16 (12.5%)	8 (10.3%)	11 (9.9%)	13 (17.3%)
Moderate/high risk for other substance use^h^ (*n* = 689)		63 (9.1%)	10 (5.1%)	10 (10.0%)	8 (6.3%)	4 (5.1%)	27 (24.3%)	4 (5.3%)
Moderate/high risk for alcohol and/or substance use^i^ (*n* = 689)		106 (15.4%)	12 (6.1%)	18 (18.0%)	19 (14.8%)	11 (14.1%)	31 (27.9%)	15 (20.0%)
Any potentially traumatic events experienced/witnessed^j^ (*n* = 693)		468 (67.5%)	73 (37.1%)	83 (83.0%)	109 (85.2%)	73 (89.0%)	81 (73.0%)	49 (65.3%)
Anxiety symptoms^k^ (*n* = 689)	Moderate or severe	70 (10.2%)	12 (6.1%)	11 (11.0%)	11 (8.6%)	0	29 (26.1%)	7 (9.3%)
Depression symptoms^l^ (*n* = 693)	Moderate or severe	71 (10.2%)	11 (5.6%)	16 (16.0%)	9 (7.0%)	5 (6.1%)	22 (19.8%)	8 (10.7%)
Anxiety and/or depression symptoms (*n* = 693)	Moderate or severe	106 (15.3%)	17 (8.6%)	21 (21.0%)	16 (12.5%)	5 (6.1%)	36 (32.4%)	11 (14.7%)
Viral load (*n* = 680)	Undetectable^m^	457 (67.2%)	143 (73.0%)	44 (47.8%)	93 (72.7%)	48 (61.5%)	74 (66.7%)	55 (73.3%)
	Suppressed, detectable < 200 copies/ml	78 (11.5%)	19 (9.7%)	33 (35.9%)	0	9 (11.5%)	10 (9.0%)	7 (9.3%)
	Suppressed, detectable 200-1,000 copies/ml	47 (6.9%)	9 (4.6%)	4 (4.3%)	17 (13.3%)	8 (10.3%)	7 (6.3%)	2 (2.7%)
	Unsuppressed, detectable > 1,000 copies/ml	98 (14.4%)	25 (12.8%)	11 (12.0%)	18 (14.1%)	13 (16.7%)	20 (18.0%)	11 (14.7%)
ART regimen at enrollment (*n* = 687)	INSTI-based	490^n^ (71.3%)	152 (77.2%)	87 (87.0%)	121 (94.5%)	62 (74.7%)	50 (46.7%)	18 (25.0%)
	PI-based	119^o^ (17.3%)	5 (2.5%)	2 (2.0%)	1 (0.8%)	5 (6.0%)	28 (26.2%)	37 (51.4%)
	NNRTI-based	78 (11.4%)	40 (23.3%)	11 (11.0%)	6 (4.7%)	16 (19.3%)	29 (27.1%)	17 (23.6%)

Regions and number of participants per country: EA East Africa (Kenya = 197), SA Southern Africa (Zambia = 50, South Africa = 50), CA Central Africa (Rwanda = 128), WA Western Africa (Cote d’Ivoire = 83), CN Caribbean, Central and South America Network (Honduras = 30, Haiti = 21, Brazil = 60), AP Asia-Pacific (Thailand = 55, Philippines = 20)

ART antiretroviral therapy, INSTI integrase strand transfer inhibitor, PI protease inhibitor, NNRTI non-nucleoside reverse transcriptase inhibitor

^a^ Assigned at birth; b Assessed using the ICAMP adherence monitoring tool, questions 4–10 (any non-zero score=positive screen); c ICAMP tool, questions 9 & 10 (any doses reported as missing); d Assessed using the ‘Multidimensional Scale of Perceived Social Support’ (MSPSS) tool, scoring < 3 (low) vs. 3–5 (moderate) vs. > 5 (high); e Assessed using the ‘Stigma in AIDS Family Inventory’ (SAFI) tool (non-zero score=positive screen); f Assessed using the AUDIT tool, scoring ≥ 8 indicates strong likelihood of hazardous/harmful alcohol consumption; g Assessed using the WHO ASSIST tool, scoring > 10; h Assessed using the WHO ASSIST tool, scoring > 3 (alcohol, tobacco and hookah use were excluded); i Excluding tobacco and hookah use; j Assessed using the ‘Life Event Checklist’ (LEC-5) tool (non-zero score=positive screen); k Assessed using the ‘Generalized Anxiety Disorder’ (GAD-7) tool, scoring ≥ 10; l Assessed using the ‘Patient Health Questionnaire’ (PHQ-9) tool, scoring ≥ 10; m For results reported as undetectable, thresholds for undetectable VL (copies/ml) included: <20 (18%); <40 (46%), < 60 (6%), < 100 (5%), < 200 (22%) and < 840 (2%); n Of these 490, 13 also had PI prescribed, 1 also had NNRTI prescribed, and 1 had both PI and NNRTI prescribed; o Of these 119, 3 also had NNRTIs prescribed

Findings on alcohol, tobacco and other substance use (self-reported and point-of-care urine test results), stratified by region, are shown in Table 2. Prior alcohol use (‘ever used’) was common (47%), whereas prior use of other substances was less commonly reported: cannabis (12%), tobacco (11%), sedatives/benzodiazepines (3.9%), cocaine (1.9%), amphetamines (1.6%), inhalants (1.5%), opioids (1.3%), hallucinogens (1.3%). Three participants reported ever having used a substance by injection. Moderate/high-risk alcohol use and other substance use in the previous 3 months was reported in 9.1% and 9.1% of YLHIV, respectively; 15% overall (*n* = 106/689) reported moderate/high-risk alcohol and/or substance use (Table 1).

**Table 2:** Self-reported and point-of-care urinary test results for alcohol and substances among youth living with HIV

Ever used		Total	EA	SA	CA	WA	CN	AP
	Alcohol (*n* = 689)	326 (47.3%)	52 (26.4%)	53 (53.0%)	54 (42.2%)	46 (59.0%)	71 (64.0%)	50 (66.7%)
	Tobacco (*n* = 689)	119 (17.3%)	6 (3.1%)	19 (19.0%)	17 (13.3%)	27 (34.6%)	34 (30.6%)	16 (21.3%)
	Cannabis (*n* = 689)	81 (11.8%)	15 (7.6%)	17 (17.0%)	9 (7.0%)	5 (6.4%)	31 (27.9%)	4 (5.3%)
	Sedatives/benzodiazepines (*n* = 688)	27 (3.9%)	1 (0.5%)	0	2 (1.6%)	0	21 (18.9%)	3 (4.0%)
	Cocaine (*n* = 689)	13 (1.9%)	1 (0.5%)	0	1 (0.8%)	0	10 (9.0%)	1 (1.4%)
	Amphetamines (*n* = 688)	11 (1.6%)	0	0	2 (1.6%)	3 (3.9%)	3 (2.7%)	3 (4.0%)
	Inhalants(*n* = 689)	10 (1.5%)	4 (2.0%)	0	0	0	5 (4.6%)	1 (1.3%)
	Hallucinogens (*n* = 688)	9 (1.3%)	0	0	0	2 (2.6%)	6 (5.4%)	1 (1.4%)
	Opioids (*n* = 689)	9 (1.3%)	1 (0.5%)	3 (3.0%)	2 (1.6%)	1 (1.3%)	0	2 (2.7%)
	Other^a^ (*n* = 683)	3 (0.4%)	1 (0.5%)	2 (2.1%)	0	0	0	0
If ever used any substance above, then ever used any drug by injection (*n* = 345)		3 (0.9%)	2 (3.9%)	1 (1.7%)	0	0	0	0
Urinary POC test positive for alcohol (*n* = 673)		46 (6.8%)	1 (1.3%)	18 (14.1%)	12 (11.2%)	7 (3.8%)	6 (6.1%)	2 (2.5%)
Urinary POC test positive for cannabis (*n* = 667)		32 (4.8%)	0	2 (1.6%)	13 (12.3%)	7 (3.8%)	10 (10.4%)	0
Urinary POC test positive for benzodiazepines (*n* = 657)		13 (2.0%)	0	0	5 (5.2%)	6 (3.2%)	2 (2.1%)	0
Urinary POC test positive for cocaine (*n* = 668)		2 (0.3%)	0	0	2 (1.9%)	0	0	0
Urinary POC test positive for amphetamines (*n* = 671)		3 (0.4%)	0	0	0	0	1 (1.0%)	2 (2.6%)
Urinary POC test positive for opioids (*n* = 654)		0	0	0	0	0	0	0
Urinary POC test positive for barbiturates (*n* = 13)		1 (0.8%)	n/a	n/a	1 (0.8%)	n/a	n/a	n/a
Urinary POC test positive for phencyclidine (PCP) (*n* = 13)		1 (0.8%)	n/a	n/a	1 (0.8%)	n/a	n/a	n/a

		Alcohol (*n* = 325)	Tobacco (*n* = 119)	Cannabis (*n* = 81)	Sedatives (*n* = 27)	Cocaine (*n* = 13)	Amphetamines (*n* = 11)	Inhalants (*n* = 10)	Hallucinogens (*n* = 9)	Opioids (*n* = 9)
If ever used, frequency in last 3 months	Never	113 (34.8%)	53 (44.5%)	34 (42.0%)	11 (40.7%)	7 (53.8%)	6 (54.5%)	6 (60.0%)	5 (55.6%)	6 (66.7%)
	Once/twice	116 (35.7%)	26 (21.8%)	17 (21.0%)	7 (25.9%)	2 (15.4%)	3 (27.3%)	1 (10.0%)	4 (44.4%)	3 (33.3%)
	Monthly	53 (16.3%)	7 (5.9%)	9 (11.1%)	3 (11.1%)	1 (7.7%)	0	1 (10.0%)	0	0
	Weekly	39 (12.0%)	10 (8.4%)	8 (9.9%)	1 (2.7%)	0	2 (18.2%)	0	0	0
	Daily or almost daily	4 (1.2%)	23 (19.3%)	13 (16.0%)	5 (18.5%)	3 (23.1%)	0	2 (20.0%)	0	0
If ever used, substance use risk score^b^	Low	262 (80.6%)	56 (47.1%)	35 (43.2%)	15 (55.6%)	8 (61.5%)	6 (54.5%)	6 (60.0%)	9 (100.0%)	7 (77.8%)
	Moderate	59 (18.2%)	58 (48.7%)	43 (53.1%)	12 (44.4%)	3 (23.1%)	5 (45.5%)	2 (20.0%)	0	2 (22.2%)
	High	4 (1.2%)	5 (4.2%)	3 (3.7%)	0	2 (15.4%)	0	2 (20.0%)	0	0

EA East Africa, SA Southern Africa, CA Central Africa, WA Western Africa, CN Caribbean, Central and South America Network, AP Asia-Pacific, POC point-of-care

^a^Hookah pipe (substance not specified)

^b^Assessed using the WHO ASSIST tool; for alcohol scoring 11–26 (moderate) and > 26 (high); for all other substances scoring 4–26 (moderate) and > 26 (high)

**Table 3:** Univariable logistic regression models assessing factors associated with (A) self-reported adherence difficulties (any missed doses) and (B) unsuppressed viral load (> 1,000 copies/ml)

			(A) Adherence difficulties (self-reported, any missed doses)^a^		(B) VL > 1,000 copies/ml	
			OR (95% CI)	*p*	OR (95% CI)	*p*
VL > 1,000 copies/ml			2.60 (1.65–4.11)	< 0.001	n/a	
Self-reported difficulties with medication adherence, assessed by	Any missed doses in the preceding 7 days or month^a^		n/a		2.60 (1.65–4.11)	< 0.001
	≥ 2 days with missed doses in the last 7 days		n/a		4.25 (2.67–6.76)	< 0.001
	≥ 4 days with missed doses in the last 7 days		n/a		11.22 (5.07–24.84)	< 0.001
Female sex vs. male			0.91 (0.68–1.23)	0.56	0.74 (0.48–1.14)	0.18
Perinatal mode of HIV acquisition (vs. non-perinatal mode)			1.28 (0.86–1.91)	0.22	2.28 (1.12–4.68)	0.02
Age at enrollment (years), centred at 15 years			1.01 (0.95–1.06)	0.83	0.97 (0.90–1.06)	0.53
Age at enrollment (years)		15–17	Ref		Ref	
		18–19	1.39 (0.92–2.12)	0.12	1.04 (0.57–1.87)	0.91
		20–21	1.04 (0.67–1.60)	0.87	0.87 (0.46–1.64)	0.66
		22–25	1.07 (0.71–1.61)	0.75	0.89 (0.49–1.62)	0.71
Ever been homeless			1.40 (0.83–2.34)	0.21	1.55 (0.81–2.97)	0.18
Ever been imprisoned			2.03 (0.97–4.29)	0.06	0.88 (0.30–2.58)	0.82
Potentially traumatic events experienced/witnessed^b^			1.88 (1.36–2.60)	< 0.001	1.12 (0.70–1.78)	0.64
Social support perceived as low (vs. moderate/high)^c^			1.68 (0.95–2.97)	0.07	1.39 (0.67–2.86)	0.37
Stigma/discrimination experienced^d^			1.70 (1.17–2.47)	0.01	1.20 (0.69–2.08)	0.52
Anxiety symptoms moderate/severe (vs. none/mild)^e^			1.99 (1.19–3.33)	0.01	1.17 (0.59–2.32)	0.66
Depression symptoms moderate/severe (vs. none/mild)^f^			1.78 (1.08–2.95)	0.03	1.56 (0.83–2.93)	0.17
Anxiety/depression symptoms moderate/severe (vs. none/mild)			1.96 (1.28–3.01)	0.002	1.19 (0.67–2.11)	0.55
Hazardous/harmful alcohol consumption^g^			2.40 (1.38–4.17)	0.002	1.17 (0.61–2.27)	0.63
Moderate/high risk for alcohol/substance use			2.06 (1.34–3.16)	0.001	1.22 (0.69–2.15)	0.50
Moderate/high risk for cannabis use^j^			3.14 (1.60–6.17)	0.001	1.84 (0.88–3.86)	0.11
Positive urinary alcohol test (vs. negative)			1.81 (0.98–3.37)	0.06	1.47 (0.69–3.16)	0.32
Positive urinary cannabis test (vs. negative)			1.74 (0.83–3.61)	0.14	2.22 (0.96–5.14)	0.06
Positive urinary amphetamine test (vs. negative)			2.05 (0.18–22.70)	0.56	2.95 (0.26–32.83)	0.38
Positive urinary benzodiazepine test (vs. negative)			1.22 (0.41–3.67)	0.73	1.08 (0.24–4.95)	0.92
ART regimen at enrollment		INSTI-based	Ref		Ref	
		PI-based	0.85 (0.57–1.27)	0.42	3.28 (2.01–5.35)	< 0.001
		NNRTI-based	0.49 (0.30–0.80)	0.01	1.06 (0.50–2.25)	0.88

VL viral load, OR odds ratio, CI confidence interval, ART antiretroviral therapy, INSTI integrase strand transfer inhibitor, PI protease inhibitor, NNRTI non-nucleoside reverse transcriptase inhibitor

^a^Assessed using the ICAMP adherence monitoring tool, questions 9 & 10 (any doses reported as missing)

^b^Assessed using the ‘Life Event Checklist’ (LEC-5) tool (non-zero score=positive screen)

^c^Assessed using the ‘Multidimensional Scale of Perceived Social Support’ (MSPSS) tool, scoring < 3 (low) vs. 3–5 (moderate) vs. > 5 (high)

^d^Assessed using the ‘Stigma in AIDS Family Inventory’ (SAFI) tool (non-zero score=positive screen)

^e^Assessed using the ‘Generalized Anxiety Disorder’ (GAD-7) tool, scoring ≥ 10

^f^Assessed using the ‘Patient Health Questionnaire’ (PHQ-9) tool, scoring ≥ 10

^g^Assessed using the AUDIT tool, scoring ≥ 8 indicates strong likelihood of hazardous/harmful alcohol consumption

^h^Assessed using the WHO ASSIST tool, scoring > 10

^i^Assessed using the WHO ASSIST tool, scoring > 3 (alcohol, tobacco and hookah use were excluded)

^j^Assessed using the WHO ASSIST tool, scoring > 3

**Table 4:** Univariable logistic regression models assessing factors associated with (A) moderate/high risk alcohol use and (B) moderate/high risk substance use

		(A) Moderate/high risk alcohol use^a^		(B) Moderate/high risk other substance use^b^	
		OR (95% CI)	*P*	OR (95% CI)	*p*
Any self-reported general difficulties with medication adherence^c^		6.41 (1.98–20.74)	0.002	6.41 (1.98–20.74)	0.002
Female sex vs. male		0.52 (0.31–0.89)	0.02	0.49 (0.28–0.83)	0.01
Perinatal mode of HIV acquisition (vs. non-perinatal mode)		0.31 (0.18–0.54)	< 0.001	0.63 (0.34–1.17)	0.14
Age at enrollment (years), centred at 15 years		1.20 (1.08–1.33)	< 0.001	1.12 (1.02–1.24)	0.02
Age at enrollment (years)	15–17	Ref		Ref	
	18–19	2.92 (1.12–7.60)	0.03	2.33 (0.93–5.81)	0.07
	20–21	2.75 (1.03–7.36)	0.04	3.50 (1.44–8.53)	0.01
	22–25	4.33 (1.74–10.81)	0.002	2.74 (1.13–6.65)	0.03
Ever been homeless		1.95 (0.94–4.05)	0.07	0.81 (0.31–2.10)	0.67
Ever been imprisoned		2.47 (0.98–6.26)	0.06	4.45 (1.96–10.11)	< 0.001
Potentially traumatic events experienced/witnessed^e^		3.07 (1.49–6.34)	0.002	3.07 (1.49–6.34)	0.002
Social support perceived as low (vs. moderate/high)^f^		0.78 (0.27–2.24)	0.65	0.78 (0.27–2.24)	0.65
Stigma/discrimination experienced^g^		1.61 (0.78–3.35)	0.20	4.16 (1.48–11.65)	0.01
Anxiety symptoms moderate/severe (vs. none/mild)^h^		1.12 (0.49–2.56)	0.79	2.91 (1.51–5.59)	0.001
Depression symptoms moderate/severe (vs. none/mild)^i^		2.85 (1.48–5.48)	0.002	2.26 (1.14–4.48)	0.02
Region	EA	Ref		Ref	
	WA	5.51 (1.61–18.88)	0.01	1.01 (0.31–3.32)	0.99
	CA	6.89 (2.25–21.13)	0.001	1.25 (0.48–3.25)	0.65
	SA	5.96 (1.85–19.25)	0.003	2.08 (0.83–5.17)	0.12
	AP	10.12 (3.18–32.16)	< 0.001	1.05 (0.32–3.47)	0.93
	CN	5.31 (1.65–17.09)	0.01	6.01 (2.78–12.98)	< 0.001
ART regimen at enrollment	INSTI-based	Ref		Ref	
	PI-based	0.88 (0.43–1.79)	0.72	1.49 (0.80–2.78)	0.22
	NNRTI-based	0.96 (0.42–2.20)	0.92	0.72 (0.27–1.87)	0.49

OR odds ratio, CI confidence interval, EA East Africa, SA Southern Africa, CA Central Africa, WA Western Africa, CN Caribbean, Central and South America Network, AP Asia-Pacific, ART antiretroviral therapy, INSTI integrase strand transfer inhibitor, PI protease inhibitor, NNRTI non-nucleoside reverse transcriptase inhibitor

^a^Assessed using the WHO ASSIST tool, scoring > 10

^b^Assessed using the WHO ASSIST tool, scoring > 3 (alcohol, tobacco and hookah use were excluded)

^c^Assessed using the ICAMP adherence monitoring tool, questions 4–10 (any non-zero score=positive screen)

^d^ICAMP tool, questions 9 & 10 (any doses reported as missing)

^e^Assessed using the ‘Life Event Checklist’ (LEC-5) tool (non-zero score=positive screen)

^f^Assessed using the ‘Multidimensional Scale of Perceived Social Support’ (MSPSS) tool, scoring < 3 (low) vs. 3–5 (moderate) vs. > 5 (high)

^g^Assessed using the ‘Stigma in AIDS Family Inventory’ (SAFI) tool (non-zero score=positive screen)

^h^Assessed using the ‘Generalized Anxiety Disorder’ (GAD-7) tool, scoring ≥ 10

^i^Assessed using the ‘Patient Health Questionnaire’ (PHQ-9) tool, scoring ≥ 10

**Table 5:** Multivariable logistic regression models assessing associations between moderate/high risk (i) alcohol use and (ii) other substance use with (A) self-reported adherence difficulties (any missed doses in the preceding 7 days or month), (B) unsuppressed viral load (> 1,000 copies/ml) and (C) detectable viral load > 200 copies/ml

		(A) Self-reported adherence difficulties (*n* = 681)		(B) Viral load > 1,000 copies/ml (*n* = 667)		(C) Viral load > 200 copies/ml (*n* = 667)	
		aOR (95% CI)	*p*	aOR (95% CI)	*P*	aOR (95% CI)	*p*
(i) Moderate/high-risk alcohol use		2.37 (1.28–4.39)	0.01	0.80 (0.34–1.88)	0.61	0.97 (0.48–1.95)	0.92
Female sex (vs. male)		0.84 (0.61–1.17)	0.30	0.82 (0.52–1.29)	0.40	0.92 (0.62–1.35)	0.67
Perinatal mode of HIV acquisition (vs. non-perinatal mode)		1.07 (0.65–1.75)	0.80	1.75 (0.80–3.82)	0.16	2.40 (1.21–4.76)	0.01
Age at study enrollment (years)		0.99 (0.92–1.06)	0.71	0.97 (0.88–1.06)	0.72	1.01 (0.93–1.09)	0.89
ART regimen at enrollment	INSTI-based	Ref		Ref		Ref	
	PI-based	1.07 (0.68–1.67)	0.77	3.21 (1.87–5.52)	< 0.001	2.45 (1.50-4.00)	< 0.001
	NNRTI-based	0.68 (0.36–1.27)	0.22	1.18 (0.48–2.85)	0.72	0.91 (0.41–2.03)	0.82
		aOR (95% CI)	p	aOR (95% CI)	P	aOR (95% CI)	p
(ii) Moderate/high-risk other substance use		2.13 (1.18–3.84)	0.01	1.62 (0.79–3.30)	0.19	1.23 (0.63–2.39)	0.54
Female sex (vs. male)		0.85 (0.61–1.17)	0.32	0.85 (0.54–1.35)	0.50	0.93 (0.63–1.37)	0.71
Perinatal mode of HIV acquisition (vs. non-perinatal mode)		1.02 (0.62–1.66)	0.95	1.84 (0.84–4.03)	0.13	2.45 (1.24–4.84)	0.01
Age at study enrollment (years)		0.99 (0.93–1.06)	0.85	0.97 (0.88–1.06)	0.45	1.00 (0.93–1.09)	0.91
ART regimen at enrollment	INSTI-based	Ref		Ref		Ref	
	PI-based	1.05 (0.67–1.64)	0.83	3.22 (1.87–5.55)	< 0.001	2.45 (1.50-4.00)	< 0.001
	NNRTI-based	0.69 (0.37–1.29)	0.24	1.25 (0.51–3.04)	0.62	0.93 (0.42–2.08)	0.87

All models were adjusted for geographical region

aOR adjusted odds ratio, CI confidence interval, ART antiretroviral therapy, INSTI integrase strand transfer inhibitor, PI protease inhibitor, NNRTI non-nucleoside reverse transcriptase inhibitor

Urine testing was positive for alcohol (*n* = 46/673; 6.8%), cannabis (*n* = 32/667; 4.8%), benzodiazepines (*n* = 13/657; 2.0%), amphetamines (*n* = 3/671; 0.4%), and cocaine (*n* = 2/668; 0.3%). Of YLHIV with positive urine tests, 37% reported no alcohol use (*n* = 17/46), 38% no cannabis use (*n* = 12/32), 100% no benzodiazepine use (*n* = 13/13), 67% no amphetamine use(*n* = 2/3), and 50% no cocaine use (*n* = 1/2).

In unadjusted analyses (Table 3A), self-reported adherence difficulties (missed medication doses in the previous 7 days and/or month) were significantly associated with unsuppressed VL, moderate/high risk alcohol and/or substance use (including cannabis use specifically), exposure to potentially traumatic events, stigma, symptoms of anxiety and/or depression, and ART regimen. Unsuppressed VL was significantly associated with perinatally-acquired HIV, self-reported missed doses, and ART regimen (Table 3B).

In unadjusted analyses, moderate/high-risk alcohol use was significantly associated with older age, male sex, exposure to potentially traumatic events, symptoms of depression, self-reported adherence difficulties, and geographical region, and was less likely among those with perinatally-acquired HIV (Table 4A). Moderate/high-risk other substance use was significantly associated with older age, male sex, exposure to potentially traumatic events, previous imprisonment, HIV-related stigma/discrimination experiences, symptoms of depression and anxiety, self-reported adherence difficulties, and geographical region (Table 4B).

In separate multivariable analyses, moderate/high-risk alcohol and other substance use were both significantly associated with self-reported adherence difficulties (adjusted odds ratio(aOR) 2.37, 95% Confidence Interval [CI] 1.28–4.39; and aOR 2.13, 95% CI 1.18–3.84, respectively) but not with unsuppressed VL (aOR 0.80, 95% CI 0.34–1.88; and aOR 1.62, 95% CI 0.79–3.30, respectively) (Table 5A and 5B).

 In sensitivity analyses using alternative definitions of self-reported adherence difficulties (Table 6), moderate/high-risk alcohol use was no longer significantly associated with adherence difficulties when higher thresholds for missed doses were applied. In contrast, moderate/high-risk use of other substances remained significantly associated with adherence difficulties, with larger effect estimates observed as the number of missed days increased. Specifically, other substance use was associated with both ≥ 2 days with missed doses (aOR 2.21, 95% CI 1.22–4.02) and ≥ 4 days with missed doses (aOR 3.00, 95% CI 1.17–7.71).

**Table 6:** Multivariable logistic regression models assessing associations between moderate/high risk (i) alcohol use and (ii) other substance use with self-reported adherence difficulties using alternative thresholds based on missed doses over the preceding 7 days

		(A) ≥ 2 days with missed doses (*n* = 665)		(B) ≥ 4 days with missed doses (*n* = 593)	
		aOR (95% CI)	*p*	aOR (95% CI)	*P*
(i) Moderate/high-risk alcohol use		1.67 (0.91–3.09)	0.10	0.93 (0.25–3.40)	0.91
Female sex (vs. male)		0.90 (0.60–1.34)	0.59	1.10 (0.50–2.41)	0.81
Perinatal mode of HIV acquisition (vs. non-perinatal mode)		0.71 (0.39–1.28)	0.25	0.89 (0.29–2.71)	0.84
Age at study enrollment (years)			0.21	1.11 (0.94–1.31)	0.23
ART regimen at enrollment	INSTI-based	Ref		Ref	
	PI-based	1.67 (1.00-2.80)	0.05	1.72 (0.70–4.18)	0.23
	NNRTI-based	0.53 (0.23–1.21)	0.13	n/a	
		aOR (95% CI)	p	aOR (95% CI)	P
(ii) Moderate/high-risk other substance use		2.21 (1.22–4.02)	0.01	3.00 (1.17–7.71)	0.02
Female sex (vs. male)		0.92 (0.61–1.37)	0.67	1.19 (0.54–2.62)	0.66
Perinatal mode of HIV acquisition (vs. non-perinatal mode)		0.71 (0.39–1.27)	0.25	0.99 (0.33–2.98)	0.98
Age at study enrollment (years)		0.95 (0.88–1.03)	0.24	1.11 (0.94–1.31)	0.23
ART regimen at enrollment	INSTI-based	Ref		Ref	
	PI-based	1.64 (0.98–2.76)	0.06	1.76 (0.72–4.33)	0.22
	NNRTI-based	0.55 (0.24–1.27)	0.16	n/a	

All models were adjusted for geographical region

OR adjusted odds ratio, CI confidence interval, ART antiretroviral therapy, INSTI integrase strand transfer inhibitor, PI protease inhibitor, NNRTI non-nucleoside reverse transcriptase inhibitor

YLHIV receiving protease inhibitor-based ART regimens were more likely to have unsuppressed VL compared to those receiving INSTI-based regimens. In sensitivity analyses, when using a different VL threshold (VL > 200 instead of > 1,000 copies/ml), findings were largely similar, except that detectable VL > 200 copies /ml was more likely among those with perinatally-acquired HIV (Table 5C).

## Discussion

Our study demonstrates the complexity between multiple factors that can influence adherence among YLHIV. Substance use among this cohort of YLHIV was relatively common, particularly use of alcohol, tobacco and cannabis. In other studies, suboptimal adherence to ART has been found with the use of alcohol, tobacco and cannabis - the three most commonly used substances among youth [7, 24–26, 46, 47]. Our findings are consistent with studies from other settings [2, 18, 26, 45]. Similar patterns of experimentation have also been observed in other studies, with higher rates of substance use occurring in older adolescents and males [2, 15, 16, 45]. 

Based on the proportions of YLHIV with positive urine tests, but reporting no alcohol/substance use, true prevalence of alcohol/substance use is likely higher than what was reported. Self-reported, recent moderate/high risk alcohol or other substance use (largely cannabis) in this cohort of YLHIV was 15% overall and was associated with self-reported adherence difficulties. Although not significantly associated with unsuppressed VL, this may be due to sample size limitations. Of those who reported recent alcohol and cannabis use, risk was moderate/high among 19% and 57% of users, respectively, indicating that a substantial proportion may benefit from intervention. Experimentation with alcohol and other substances is common during adolescence and young adulthood, including among youth without HIV. Population-based studies from many of the regions represented in this cohort report substantial levels of alcohol and substance use among young people [48], suggesting that these behaviours are not unique to youth living with HIV. However, for YLHIV, substance use and mental health challenges may have greater clinical relevance because of their potential impact on treatment adherence, engagement in care, and long-term HIV outcomes.

Moreover, our study reflects the extent of psychosocial challenges experienced by care-engaged YLHIV and demonstrates associations between substance use and psychosocial stressors, in keeping with prior multiregional studies from resource-limited settings [47]. This provides insight into the complexity of managing HIV in adolescents, and the need for multi-disciplinary teams and clinics that meet the needs of adolescents. Significant associations with depression, anxiety and potential traumatic exposures, as also described by Levy et al. [25], were found in relation to substance use in this age group. In our YLHIV cohort, 10% had features of moderate/ severe depression or moderate/severe anxiety, 68% had experienced potential traumatic events, and 79% had experienced stigma. The co-occurrence of these challenges with high rates of self-reported adherence difficulties suggests that a stronger focus is needed on mental health among YLHIV, a finding that is consistent with evidence from studies among young adults in South Africa [18]. Interventions that address mental health may positively impact substance use, ART adherence, and overall well-being, reinforcing the importance of routinely assessing these issues as part of comprehensive HIV care.

The absence of an observed association between alcohol or other substance use with viral non-suppression in this study should be interpreted in the context of a relatively care-engaged population. All participants were aware of their HIV status, enrolled in care, and had been receiving ART for at least three months prior to enrolment. Prior studies have suggested that the association between alcohol use and viral non-suppression may operate predominantly through earlier stages of the HIV care cascade, including delayed linkage to care, interruptions in ART initiation, and poor retention, rather than through direct effects on virologic control among individuals actively receiving treatment [49]. Other studies have reported persistent associations between alcohol use and virologic outcomes even among individuals on ART, highlighting heterogeneity across settings, populations and treatment eras [50]. 

Approximately half of the YLHIV in this cohort (49%) reported missing medication doses in the previous month, although only 14% had unsuppressed VL. In sensitivity analyses using alternative definitions of adherence difficulties, alcohol use was not associated with higher-frequency missed dosing, whereas other substance use showed stronger associations as the number of missed doses increased, suggesting that substances other than alcohol may be more strongly linked to clinically meaningful adherence difficulties. INSTI-based regimens were used by 71% of participants, and this class of antiretroviral is known to be highly effective at reducing VL along with having a high genetic barrier to resistance, making it more forgiving of missed doses than other regimens. Similarly, a study from Uganda hypothesised that the lack of association between alcohol use and viral suppression among individuals on ART may reflect improvements in modern ART regimens, which require lower levels of adherence to achieve and maintain viral suppression, thereby potentially attenuating the impact of alcohol use on adherence and virologic outcomes [46]. Future research should explore longitudinal associations between substance use, adherence, and VL, and evaluate targeted interventions that address the intersecting challenges of substance use and mental health in YLHIV.

### Strengths and Limitations

A strength of our study is the inclusion of a diverse cohort of YLHIV from multiple geographical regions, mostly in resource-limited settings, and the use of standardised screening tools across all sites. The use of both self-reported and objective measures of substance use strengthened the analysis of substance use, despite urine tests generally only detecting use of substances in the preceding three days.

A limitation of our study was the somewhat small sample size (relative to the prevalence of some outcomes), which contributed to wide confidence intervals. This analysis was restricted to youth who were aware of their HIV status, engaged in care, and met eligibility criteria for study enrolment. As a result, the prevalence of substance use, mental health difficulties, and adherence challenges observed here is likely to underestimate the burden among YLHIV who are disengaged from care or lost to follow-up, among whom these challenges may be more pronounced. The urine tests used were not standardised across all sites. The potential for false positive urine tests, as well as variability in test sensitivity and specificity, should also be considered. This makes assessing the true prevalence of substance use challenging. HIV drug resistance results were not available, therefore this potentially important association with unsuppressed VL could not be assessed.

## Conclusions

Use of alcohol and other substances is fairly common among YLHIV and should be addressed in the ongoing management of YLHIV. Our results show that moderate/high risk alcohol and substance use is prevalent among YLHIV across diverse regions and is associated with self-reported adherence challenges. Substance use is a social topic not often addressed in general consultation unless warning signs are present. Without in-depth questioning of psychosocial stressors, opportunities to manage such underlying challenges may be missed, which may influence treatment adherence. Our findings highlight the need for comprehensive, integrated care approaches that encompass mental health support, substance use interventions, and adherence counselling to improve health outcomes among YLHIV.
